# Comparison of Descriptor- and Fingerprint Sets in Machine Learning Models for ADME-Tox Targets

**DOI:** 10.3389/fchem.2022.852893

**Published:** 2022-06-08

**Authors:** Álmos Orosz, Károly Héberger, Anita Rácz

**Affiliations:** Plasma Chemistry Research Group, Research Centre for Natural Sciences, Budapest, Hungary

**Keywords:** QSPR, XGBoost, neural network, molecular descriptor, fingerprint

## Abstract

The screening of compounds for ADME-Tox targets plays an important role in drug design. QSPR models can increase the speed of these specific tasks, although the performance of the models highly depends on several factors, such as the applied molecular descriptors. In this study, a detailed comparison of the most popular descriptor groups has been carried out for six main ADME-Tox classification targets: Ames mutagenicity, P-glycoprotein inhibition, hERG inhibition, hepatotoxicity, blood–brain-barrier permeability, and cytochrome P450 2C9 inhibition. The literature-based, medium-sized binary classification datasets (all above 1,000 molecules) were used for the model building by two common algorithms, XGBoost and the RPropMLP neural network. Five molecular representation sets were compared along with their joint applications: Morgan, Atompairs, and MACCS fingerprints, and the traditional 1D and 2D molecular descriptors, as well as 3D molecular descriptors, separately. The statistical evaluation of the model performances was based on 18 different performance parameters. Although all the developed models were close to the usual performance of QSPR models for each specific ADME-Tox target, the results clearly showed the superiority of the traditional 1D, 2D, and 3D descriptors in the case of the XGBoost algorithm. It is worth trying the classical tools in single model building because the use of 2D descriptors can produce even better models for almost every dataset than the combination of all the examined descriptor sets.

## Introduction

The optimization of the ADME-Tox (adsorption, distribution, metabolism, excretion, and toxicity) properties of potential drug candidates plays a major role in any drug design project. Drug candidates often fail due to their suboptimal drug safety properties (Ferreira and Andricopulo, 2019; Rácz et al., 2021; Danishuddin et al., 2022). As the *in vivo* ADME-Tox experiments are expensive and time-consuming, *in silico* QSPR models are frequently used for the prediction of ADME-Tox properties with the great advantage as they can be used before the synthesis of the compounds (Yang et al., 2018).

ADME-Tox classification models with the use of medium-sized databases (more than 1,000 compounds) can help in the early phase of the drug design process, when a simple prefiltering is sufficient based on the ADME features. Machine learning algorithms are nowadays routinely applied for activity prediction (QSAR models) (Lima et al., 2016), like in other fields of science, as they can greatly increase the performance of ADME-related QSPR models, as illustrated by the relevant literature of the last decade. In our recent review, a detailed comparison of machine learning–driven ADME-Tox classification models was presented concentrated on the last 5 years. Our analysis showed that tree-based methods are the most popular choices amongst the machine learning algorithms for ADME-Tox model development (Rácz et al., 2021). The superiority of the machine learning algorithms over the traditional classification methods was also mentioned in the work of Tsou et al. (2020).

The categorization of descriptors is generally performed by dimensions (how many dimensions are used for calculations); so, one can distinguish 0-, 1-, 2-, and 3-dimensional descriptors and their combinations. Several molecular representations are used in the QSPR model building processes. Different molecular fingerprints and thousands of 1D, 2D, and 3D molecular descriptors can be generated with dedicated software and online tools (Todeschini and Consonni, 2000; Bajusz et al., 2017). Although sometimes it looks like a neglected part in the publications, which should not be optimized, it has a great importance (not just) in the ADME-Tox model building: the performance of models can highly depend on the applied input variables (Nembri et al., 2016; Rácz and Keserű, 2020).

The selection of proper descriptors is not a trivial task: consensus modeling (Abdelaziz et al., 2016; Lei et al., 2016) and even 4D QSAR models have already been developed by Ravi et al (2001) as early as in 2001. On the other hand, Doweyko (2004) emphasized the illusions of using 3D QSAR models. Also, different types of descriptors can be applied together in a hierarchical manner (Basak et al., 1997) to achieve better performance.

The descriptors are not to be used with their raw calculated (or measured) values; descriptor reduction should be carried out first, namely, constant and highly correlated descriptors are to be eliminated (Todeschini and Consonni, 2000; Rácz et al., 2019a). It is worth to mention again that the optimization, selection, or combination of different descriptor types is highly recommended for the sake of better performances. On the other hand, we have to note that there are other non-negligible aspects of the model building process to improve and develop robust QSPR models, such as the use of different validation sets (Gramatica, 2007), the use of more than one performance parameter for the models (Rácz et al., 2015; Gramatica and Sangion, 2016), or the balancing of the binary classification set (Rácz et al., 2019b).

In this study, our aim was to provide a detailed comparison of the most well-known descriptor sets for ADME-Tox model buildings on six different binary classification targets (with more than 1,000 molecules in each). Naturally, we aimed to find the optimal combinations and significant differences between their performances, too. Two well-known algorithms with superior performances, the tree-based XGBoost and the neural network-based RPropMLP, were applied for modeling, to show whether the differences (or similarities) of the five fingerprint and descriptor sets are general or carry the specificities of peculiar datasets.

## Materials and Methods

### Datasets and Their Curation

In this study, we have examined six different datasets that were previously applied for building QSAR models. The targets were the following: Ames mutagenicity (Hansen et al., 2009), P-glycoprotein inhibition (Broccatelli et al., 2011), hERG inhibition (Alves et al., 2018), hepatotoxicity (Wu et al., 2019), blood–brain-barrier permeability (Roy et al., 2019), and CYP 2C9 inhibitory activity (PubChem, 2021). The Ames mutagenicity dataset was collected by Hansen et al. (2009) and consists of 6,512 molecules from six different sources. The classification of active and inactive molecules was carried out according to the Ames test. The P-glycoprotein (P-gp or ABCB1) inhibition data comprise 1,275 molecules with IC_50_ values, collected by Broccatelli et al. (2011), based on 61 references. The hERG dataset comprises 4,787 molecules, acquired from the work of Alves et al. (2018) which was part of a comparison study between QSAR and MuDRA modeling. The hepatotoxicity dataset was gathered by Wu et al. from three public databases [SIDER (Kuhn et al., 2010), OFFSIDES (Tatonetti et al., 2012), and CTD (Davis et al., 2019)], followed by the generation of triple corresponding decoys using RApid DEcoy Retriever (RADER) (Wang et al., 2016). The resulting set contained 2,476 active and inactive molecules. The BBB permeability database of Roy et al. (2019) was obtained from four different publications, forming a set of 1,864 molecules. They distinguished between activity and inactivity according to the given logBB values. Last, for examining the cytochrome P450 2C9 isoform inhibitory activity of molecules, we used a public database from PubChem (AID = 1851). In the database, the inhibitory activity/inactivity was determined according to the NCGC Assay Protocol. The details of the datasets are shown in Table 1. The downloaded datasets were prefiltered by the authors; in the case of the AID 1851 CYP 2C9 dataset, the duplicates and molecules with inconclusive activity classes were excluded.

**TABLE 1:** Summary of the applied datasets.

Dataset	Original dataset			Final dataset			Reference
	Dataset size	Active molecule	% of actives	Dataset size	Internal set	External set	
Mutagenicity	6,512	3,503	54	6,190	4,952	1,238	Hansen et al (2009)
P-glycoprotein	1,275	666	52	1,180	944	236	Broccatelli et al (2011)
hERG	4,787	2,749	57	4,612	3,690	922	Alves et al (2018)
Hepatotoxicity	2,476	619	25	2,414	1932	482	Wu et al (2019)
BBB	1864	1,438	77	1750	1,400	350	Roy et al (2019)
CYP 2C9	12,776	5,800	45	12,379	9,904	2,475	PubChem (2021)

We have applied an additional filtering protocol for all the datasets: removing salts, filtering with the criteria of the number of heavy atoms >5, and element filter (C, H, N, O, S, P, F, Cl, Br, and I) with the open-source RDKit (www.rdkit.org) and CDK (Steinbeck et al., 2003) packages. The geometry optimization of the 3D structures for the further descriptor generation was performed with the Macromodel in the Schrödinger (Schrödinger Release 2021-3, 2021) software suite.

### Molecular Representations

Molecular descriptors can be defined simply as measured or computationally calculated values associated with a certain molecule (Bajusz et al., 2017), or according to the formulation of Todeschini and Consonni (2000): a molecular descriptor is a result of a logical and mathematical transformation, in which the chemical information is put into a number or a result of a standardized experiment. This way the chemical data can be used in a qualitative or quantitative analysis. In this study, regarding the applied methods for the representation of the investigated chemical information, five different types of molecular descriptors were employed: three different molecular fingerprints (MACCS, Atompairs, and Morgan) as well as two-dimensional (2D) and three-dimensional (3D) descriptors. The two-dimensional descriptor set contained all the one- and zero-dimensional descriptors as well.

The molecular fingerprints are projected as bit strings in most cases, although any vector of numerical values can be applied as a fingerprint in cheminformatics. MACCS (Molecular Access System by Molecular Design Limited), often referred to as the prototype of substructure key-based fingerprints, has two major types: the 166-key version and the 960-key version. The key-based fingerprints contain a group of molecular characteristics (*e.g*., atom environments) suitable for encoding the molecules.

Atompairs were first published for their application of structure–activity studies (Carhart et al., 1985). The method introduced the algorithm of atom typing, that is, computing certain values for each atom of the molecule. Using these values, each atom pair is computed: for *n* atoms, there are n*(*n*-1)/2 number of pairs.

The Morgan fingerprints (or the modified versions: extended connectivity fingerprints, ECFP) were initially established for solving problems linked to isomorphism (Morgan, 1965). This descriptor, also called a circular fingerprint, is similar to the atom pairs: it relies on the fragmentation of molecules into circular fragments, with each atom encoded as atom types. ECFP fingerprints can be considered the most well-known standard of molecular fingerprints.

The term “2D” in 2D descriptors stands for the generation method of the descriptor: it is derived from the 2D representation of the molecule. The values of the 2D descriptors are independent from the numbering of the atoms that is why they are often referred to as graph invariants (Todeschini and Consonni, 2000). Typical 2D descriptors include: 1) the different physicochemical parameters such as log*P* or p*K*_a_ values 2) or the topological descriptors, such as the Wiener or Randić branching index. (Bajusz et al., 2017). The derivation of the 3D descriptors is parallel to the 2D case, only their input is the 3D representation. It is also a big family of descriptors: it includes 1) electronic descriptors, 2) volume descriptors (such as molecular volume index and geometric volume), 3) shape descriptors, and 4) WHIM, GETAWAY, and EVA families, just to name a few.

The 2D and 3D descriptors were calculated with Dragon 7 (Kode Cheminformatics), while the three different molecular fingerprints were calculated with the open-source cheminformatics package, RDKit (www.rdkit.org). However, in the case of 2D descriptor calculations, the classical 1D and 0D descriptors were also generated (such as molecular weight and number of heteroatoms), and altogether these will be referred to as 2D descriptor sets for the sake of simplicity. The 2D and 3D molecular descriptors were filtered based on the intercorrelation limit of 0.997 (Rácz et al., 2019a). Constant descriptors were also excluded from the datasets. An additional normalization step was carried out on the molecular descriptors before the model building. The number of variables is shown for each descriptor set and for each ADME dataset in Supplementary Table S1.

### Machine Learning Algorithms

The machine learning workflows were developed using the KNIME analytics platform (4.4.0 version, KNIME GmbH, Konstanz, Germany). For building the classification models of ADME-Tox parameters, we applied two machine learning algorithms: extreme gradient boosting (XGBoost) and resilient backpropagation network (RPropMLP).

XGBoost is a scalable tree-boosting system presented by Chen and Guestrin (2016). It is a popular and effectively applied machine learning algorithm, and the roots of the method were established by Friedman (2001). The idea behind the technique is similar to the tree-based ensemble methods; however, it is extended with a boosting step. Boosting is basically a step-by-step improvement of the trees with the minimization of the error. Some of the most important factors of its success are the scalability of parameters on many levels, and its faster computation speed than other existing solutions (Sheridan et al., 2016; Zhang and Gong, 2020). Nowadays, the algorithm produces state-of-the-art solutions for a wide range of problems, especially in larger datasets. Its advantages are clearly emphasized by the fruitful applications of the method in machine learning and data mining challenges such as Kaggle competitions, for instance.

RPropMLP applies an algorithm of backpropagation learning, which is a frequently used method for supervised learning with multi-layered feed-forward networks, and means a repeated employment of a particular chain rule to compute the effect of each weight in the network. The step sizes, set individually for each weight, are independent of the value of the partial derivative, which is a main advantage of this method next to its speed and robustness (Riedmiller and Braun, 1993).

The developed KNIME workflow for the classification modeling is shown in the Supplementary Material.

### Applied Statistical Analysis and Performance Evaluation

The iterative process of the classification modeling with different input variables and algorithms has been evaluated based on 18 different performance parameters for each case study. The abbreviations for performance parameters are as follows: AUAC, AUC, AP, TPR, TNR, PPV, NPV, BM, MK, LRp, LRn, DOR, MCC, Cohen κ, ACC, BACC, Jaccard, and F1. The detailed description and equations of the parameters can be found in our previous work (Rácz et al., 2019b), and the expansions of the abbreviations are provided in the Supplementary Table S2. The performance metrics were used for the evaluation of the models with factorial ANOVA (variance analysis) for each case study separately and together as well. ANOVA can help us to detect the significant effects of the applied factor variables (the differences between the means of the factor classes). The applied factors were: 1) the descriptor sets, six levels (5 + Total), 2) the algorithms, two levels, and 3) the validation sets, three levels in the separate case study evaluation part. The list of the calculated performance parameters is provided in the Supplementary Material. The performance parameters were range-scaled (between 0 and 1) before the analysis of variance. ANOVA was carried out in STATISTICA 13 software (Tibco Software Inc., Palo Alto, United States).

## Results and Discussion

### Modeling Workflow

**FIGURE 1:**
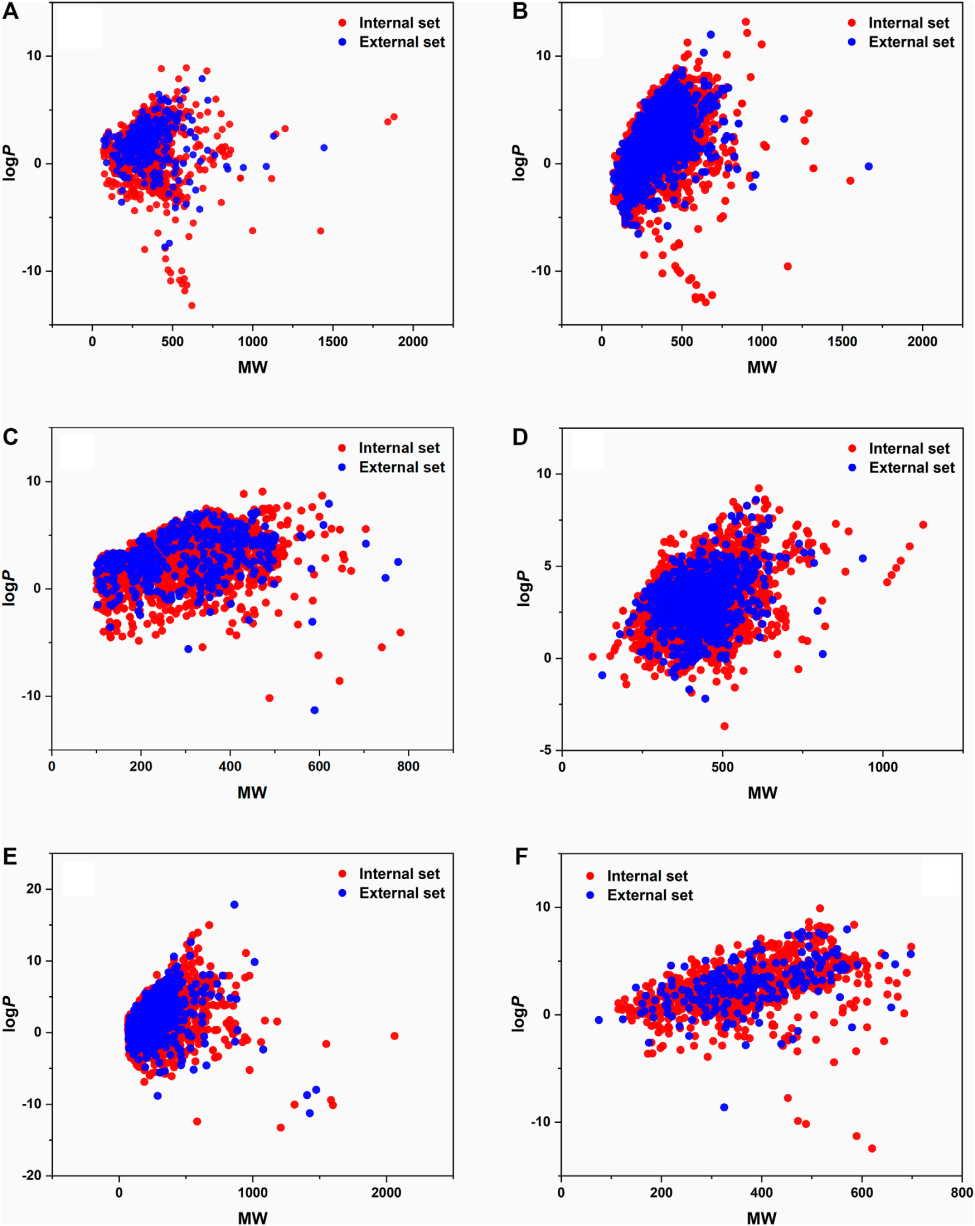
LogP values are plotted against the MW of the molecules for each dataset, internal and external sets separately. **(A)** BBB, **(B)** CYP 2C9, **(C)** hepatotoxicity, **(D)** hERG, **(E)** mutagenicity, and **(F)** P-glycoprotein datasets.

All six datasets with the different X input variables were handled with the same modeling workflow in KNIME. After importing and normalizing the variables, the datasets were split into internal and external sets. The external sets were selected with stratified sampling based on the classes and contained 20% of the whole dataset in each case. In the following parts, only the remaining 80% was used, and the external sets were applied only for the external validation of the final models. The covered space of the datasets is illustrated in the MW-logP space in Figure 1. We can conclude that the distribution between the external and internal sets is appropriate (can be considered the same). The internal and external sets are plotted separately. Moreover, *t*-distributed stochastic embedding (*t*-SNE) plots of the datasets are also presented in Supplementary Figure S1.

We have carried out the workflow with and without the application of a genetic algorithm as well. XGBoost and RPropMLP methods were used on the selected X variables and the whole datasets as well. The number of selected variables was always below 500.

An iterative process was included for the balancing of the datasets: it was necessary, especially in those cases, in which the number of actives was not around 50% in the dataset. It meant that the number of actives was fixed, and we selected the same amount of inactives from the database. The selection process was repeated ten times; thus, the inactive molecules always changed. The use of balanced datasets in binary classification can greatly affect the final outcome based on our previous study (Rácz et al., 2019b).

Another iteration step was to split the internal dataset into training/cross-validation and internal validation parts. Since the machine learning models usually provide perfect accuracies (1.0) on the training set, we omitted the collection of these data in the output files. Stratified sampling based on the classes was used with a 80:20 ratio for splitting the cross-validation (CV) and internal validation sets. The split process was repeated five times. It is important to note that there was no overlap in one iteration circle between the cross-validation and internal validation set. The complete process can be followed in detail in our KNIME workflow in the Supplementary material.

The predicted class probabilities with each algorithm were grouped by the validation sets (cross-validation, internal, and external). Mean class probabilities were calculated for each molecule. The whole process was carried out for the five different molecular descriptor sets and their merged versions. It should be noted that the genetic algorithm was not used in the case of the merged dataset because we originally wanted to see the benefit of the combined descriptors simply, as it is a very popular choice in the literature. Moreover, the number of descriptors was above 5,000, which would have really increased the calculation time in the case of the genetic algorithm.

The class probability values were used for the calculation of the 18 performance parameters (as aforesaid). The performance parameters were range-scaled before ANOVA and LRn were reversed due to their opposite direction compared to other metrics. ANOVA was carried out for the six datasets separately and in a merged version too with the use of the 18 performance parameter values. The following results are presented in a way that the target-specific results can be also concluded.

### Results of the BBB Dataset

**FIGURE 2:**
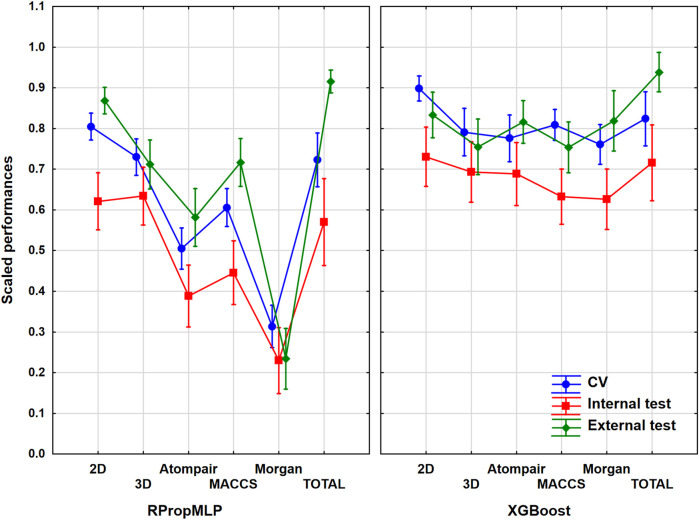
Plot of the scaled performances based on the different molecular descriptor types and different machine learning algorithms. The 18 performance parameters were range-scaled between 0 and 1 and averaged across all models with the given descriptor types/ML algorithms. The result of CV is marked with blue, the internal test result is marked with red, and the external is marked with green. The average scaled performance is plotted in each case with the 95% confidence intervals.

He have worked with 1,750 different molecules, which were split into 1,400 internal and 350 external ones. It was a mid-sized dataset, almost the smallest one out of the six. The models with and without variable selection were evaluated together in ANOVA for each descriptor set. Although, we have checked the results and the pattern was the same in all cases, the performances were slightly worse when the genetic algorithm was used. The input data for ANOVA were the range-scaled performance parameters and the three factors, namely, the descriptor types (six levels, *i.e*., six groups), the validation sets (three levels: CV, internal, and external), and the machine learning algorithms (two levels). We have to emphasize that we also analyzed the merged descriptor sets, which we will call total. The 2D descriptor set contains the 0- and 1-dimensional descriptors as well. The result is presented in Figure 2, in which the three factors are used together. ANOVA showed that the effect of the molecular descriptor sets and the machine learning algorithms are statistically significant (*α* = 0.05).

Generally, the application of 2D descriptors resulted in the best classification performances for both the RPropMLP and XGBoost algorithms in the case of the BBB dataset. The only exception is the external validation, in which the application of all descriptors (Total) resulted in better performance. When comparing the three fingerprints, we acquired the best scaled performance using the MACCS fingerprint in the case of RPropMLP, and the Atompairs performed the best when paired with the XGBoost. The Morgan fingerprint appeared to be the least effective descriptor in the case of RPropMLP, but it behaved equally well as the MACCS, when applied with the XGBoost. If we compare the performance of the two algorithms, we can see that the XGBoost algorithm produces a robust and precise outcome, since the range of the scaled performances is narrower (∼[0.60; 0.95]) and the values are generally higher than those for the RPropMLP (∼[0.23; 0.92]). Using RPropMLP, the external test set resulted in the best scaled performances for every descriptor set, except for 3D and Morgan. However, for XGBoost, the CV resulted in a better scaled performance than the external test set in case of the 2D, 3D, and MACCS descriptors. The slightly better performance of the external (or internal) test set than CV is very common in the machine learning modeling practice, and it is not connected to the overfitting of the models. The range of the scaled performance values of the three validation sets was the narrowest with the application of the 3D and Morgan descriptors. Moreover, in the case of RPropMLP, the validation sets were further away from each other compared to the XGBoost algorithm. After all, the optimal result is to be obtained using the 2D descriptors for the BBB dataset.

### Results of the CYP 2C9 Dataset

**FIGURE 3:**
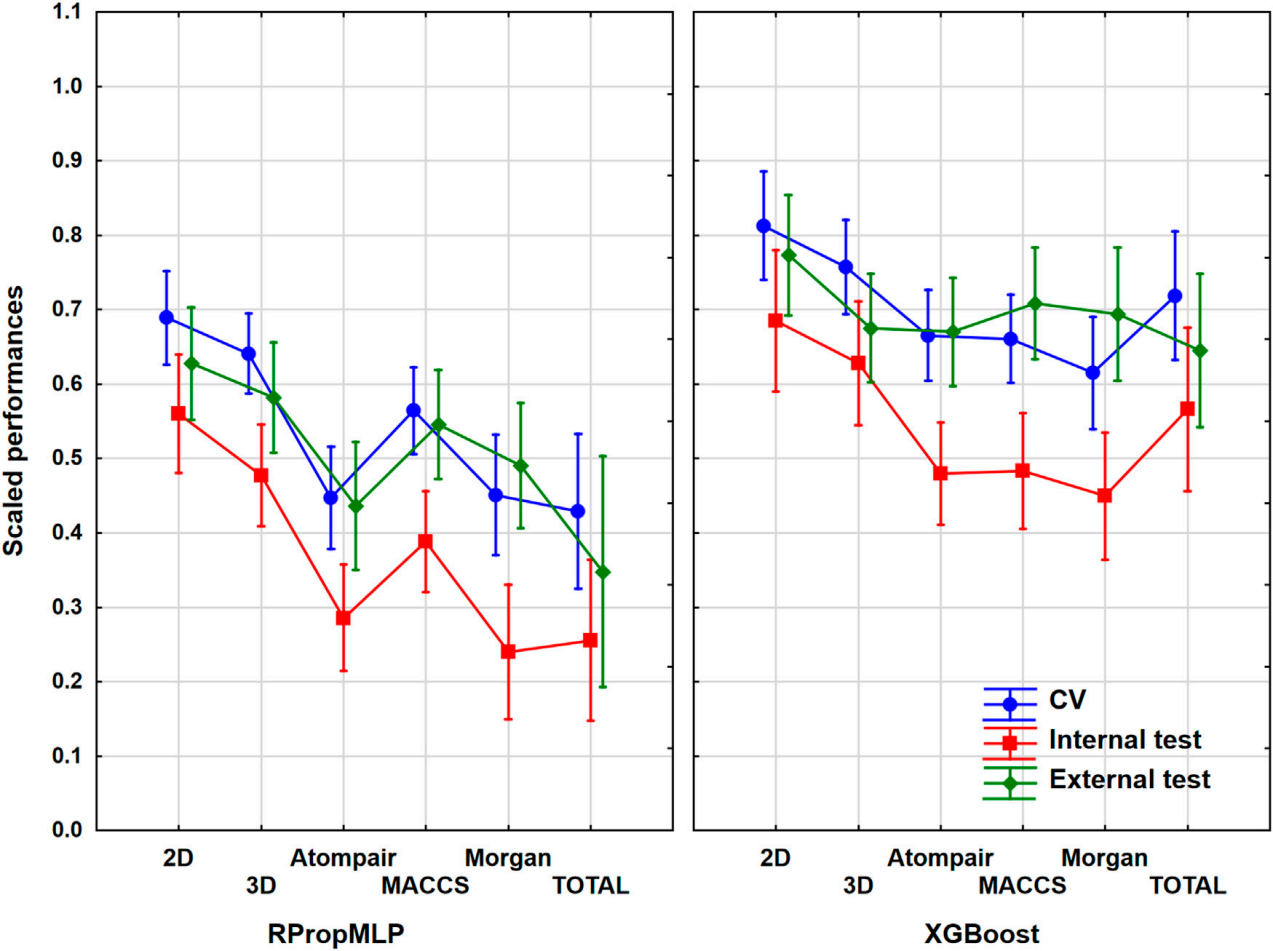
Scaled performances of the models in case of the CYP 2C9 dataset based on the molecular descriptor types and machine learning algorithms. The 18 performance parameters were range-scaled between 0 and 1 and averaged across all models with the given descriptor types/ML algorithms. The result of CV is marked with blue, the internal test result is marked with red, and the external test is marked with green. The mean is plotted in each case with the 95% confidence intervals.

The cytochrome P450 2C9 dataset was the largest one out of the six case studies, with more than 12,000 different molecules. As it was collected from the PubChem database, this case study is a frequently used one in the literature. ANOVA was performed in the same way as in the case of BBB, and the result is illustrated in Figure 3. ANOVA showed statistically significant differences (*α* = 0.05) between the six molecular descriptor sets and the two machine learning algorithms too.

In case of the CYP 2C9 dataset, the 2D descriptors also resulted in a superior scaled performance value for both algorithms, and ultimately it also brings the best classification performance similar to the BBB dataset. Comparing the fingerprint descriptors, the MACCS showed the best scaled performances combined with both algorithms, and the other two (Morgan and Atompair) presented almost identical results. When we analyze the two algorithms, the better scaled performance values are again noticeable in the XGBoost graph, although the results of XGBoost are in a lower range (∼[0.40;0.80]) in this situation than it was in the BBB dataset. If we study the validation sets, the internal test set presented the lowest scaled performances in all cases, similar to the BBB dataset (indirectly, this observation suggests that the external test set contains molecules within the applicability domain of the model, as the external validation performance was not worse than the internal). The CV and the external validation sets share the top scaled performance results, and they are overlapping in each case. The three validation sets fall in a narrower range when the 2D descriptors, the 3D descriptors, and the combined total version are applied, and it widens, when the three fingerprint descriptor sets are employed. It means that the use of classical descriptors can provide more robust models. Generally, the optimal result for the scaled performance can be obtained, when the 2D descriptor set is used for this dataset.

### Results of the Hepatotoxicity Dataset

**FIGURE 4:**
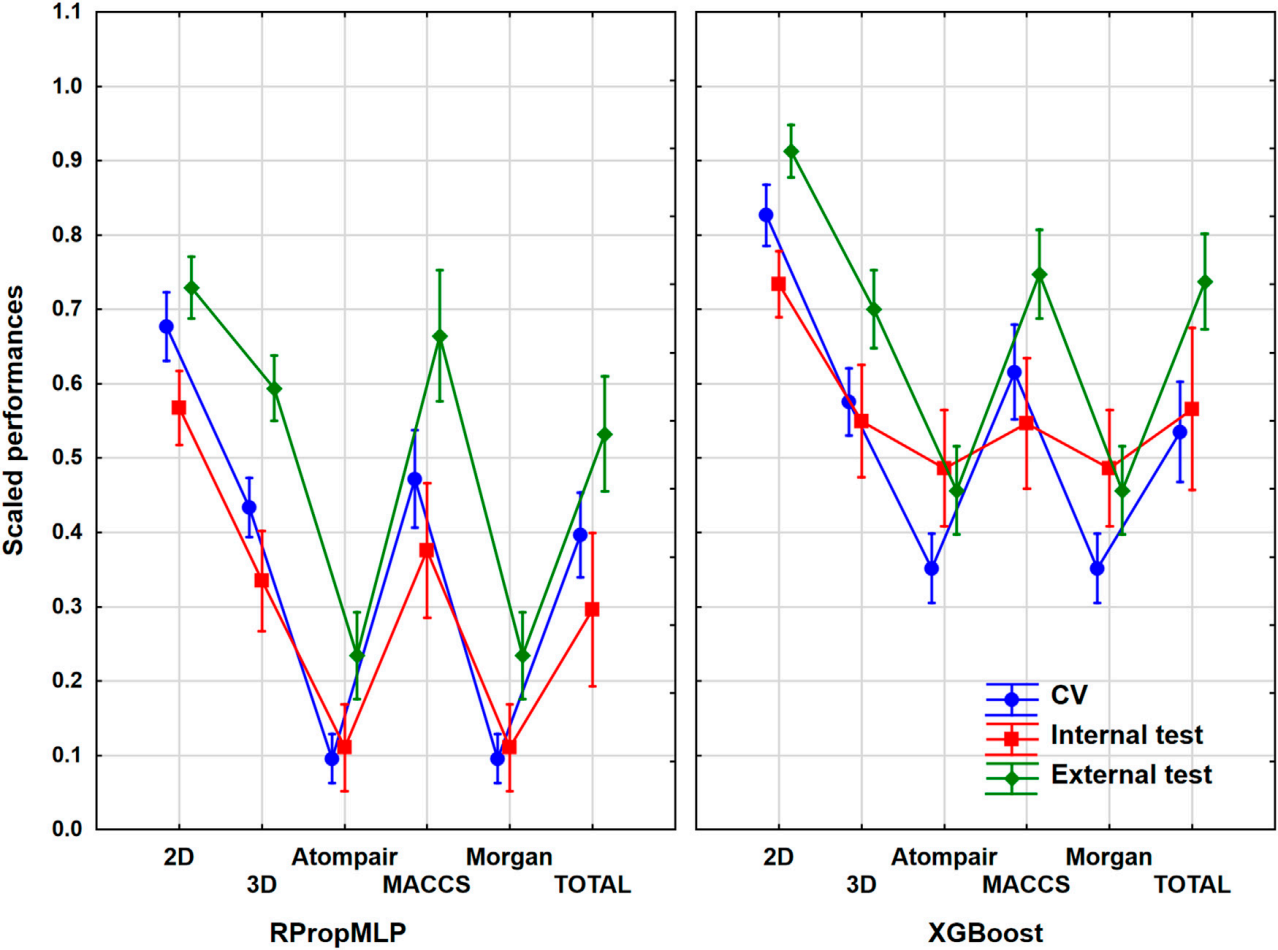
Scaled performances of the models in case of the hepatotoxicity dataset based on the molecular descriptor types and machine learning algorithms. The 18 performance parameters were range-scaled between 0 and 1 and averaged across all models with the given descriptor types/ML algorithms. The result of CV is marked with blue, the internal test result is marked with red, and the external test is marked with green. The mean of the scaled performance parameters is plotted in each case with the 95% confidence intervals.

The hepatotoxicity case study contained more than 2,400 unique molecules. In this case, the ratio of the actives was 25% in the original set before the data curation, which meant that the balancing of the dataset was really important here. ANOVA was carried out with the same protocol as in the previous cases. The most important assumptions are shown in Figure 4. ANOVA showed that the effects of the molecular descriptors and machine learning algorithms are statistically significant (*α* = 0.05).

The descriptor pool that resulted in the highest scaled performance values is the 2D descriptor set for both algorithms in case of the hepatotoxicity dataset. Among the fingerprint-type descriptors, the best performance parameter was resulted by the MACCS, while the other two fingerprints presented very low performance parameters compared to the latter fingerprint descriptors. The XGBoost performs better: the scaled performance values are significantly higher than the ones obtained with the RPropMLP. The range of the validation sets is narrow, when 2D, Atompair, and Morgan are used, and gets wider when 3D and MACCS are employed. The three validation sets are closer to each in the case of XGBoost models: these models can be considered the more robust ones. Ultimately, the optimal result is to be obtained, if the 2D descriptors are used.

### Results of the hERG Dataset

**FIGURE 5:**
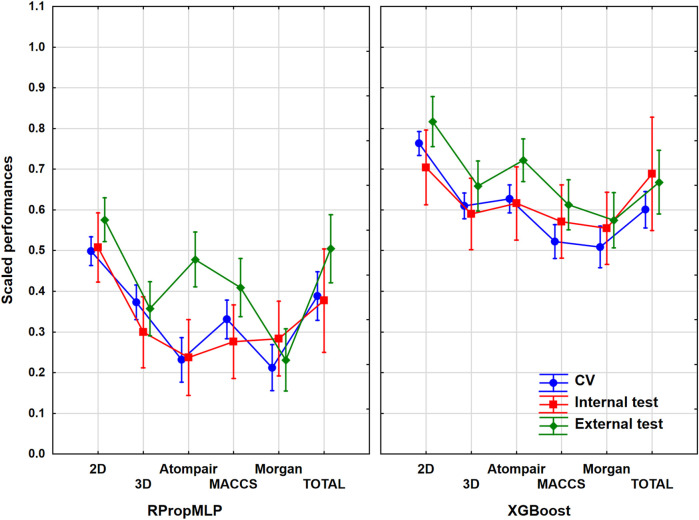
Scaled performances in the case of hERG inhibition dataset based on the molecular descriptor types and machine learning algorithms. The 18 performance parameters were range-scaled between 0 and 1 and averaged across all models with the given descriptor types/ML algorithms. The result of CV is marked with blue, the internal test result is marked with red, and the external test is marked with green. The mean of the scaled performances is plotted in each case with the 95% confidence intervals.

The hERG inhibition dataset contained more than 4,000 diverse molecules. As the inhibition of the hERG K+ channel can lead to lethal cardiac arrhythmia, this is an important ADME-Tox antitarget. The used dataset can be considered a medium-sized one, as only a few can be found with more than 10,000 molecules in the literature. The evaluation of models for this case study was carried out with the previously determined ANOVA workflow. Figure 5 shows the comparison of the molecular descriptors and machine learning algorithms for the scaled performances. ANOVA showed that statistically significant differences (*α* = 0.05) can be detected between the six different molecular descriptor sets and the two machine learning algorithms.

The modeling of the hERG dataset resulted in the best scaled performance parameters for the 2D descriptor pool: the highest values and narrowest range of the validation sets can be found at this descriptor for both algorithms. Of the fingerprint descriptors, the MACCS presented the best results (in the case of the internal set and CV) for the RPropMLP and the Atompair for the XGBoost. The XGBoost outperforms the RPropMLP in the scaled performance values likewise in all previous cases. The differences are smaller in the case of hERG models between the three validation sets, especially in the case of XGBoost models. The range of the values of the validation sets is particularly narrow, when the 2D, 3D, and Morgan descriptors are applied, and are slightly wider, when the Atompair and MACCS were combined with the RPropMLP method. Consequently, we can conclude that the 2D descriptors present the most likely optimally scaled performance values for this hERG dataset.

### Results of the Mutagenicity Dataset

**FIGURE 6:**
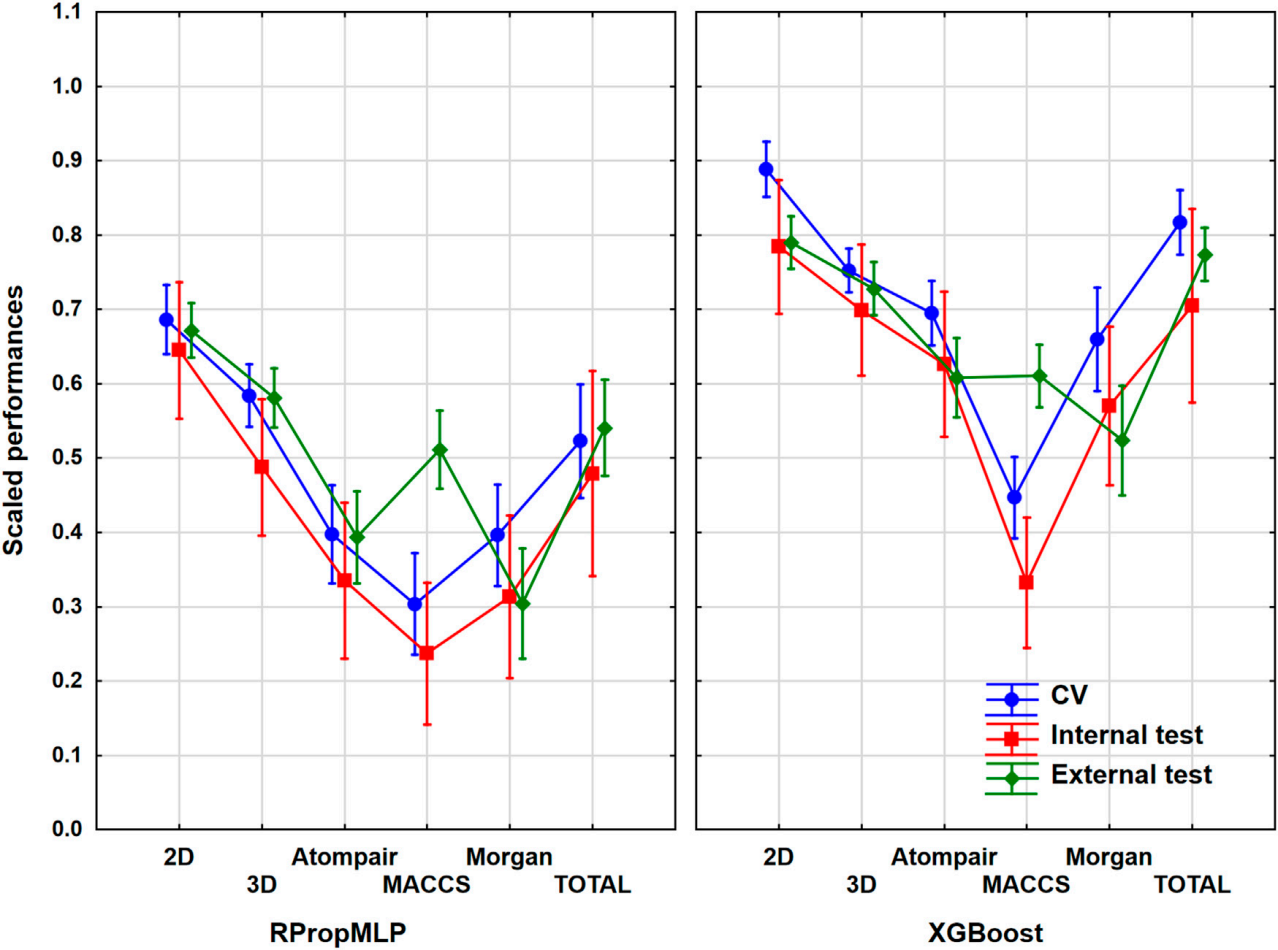
Scaled performances of the models in the case of the Ames mutagenicity dataset based on the molecular descriptor types and machine learning algorithms. The 18 performance parameters were range-scaled between 0 and 1 and averaged across all models with the given descriptor types/ML algorithms. The result of CV is marked with blue, the internal test result is marked with red, and the external test is marked with green. The mean of the scaled performance parameters is plotted in each case with the 95% confidence intervals.

The Ames mutagenicity dataset (based on the work of Hansen et al (2009)) was used as a common case study in this section. It contained more than 6,000 molecules with 54% of actives. The same workflow was applied for the evaluation of the models as previously mentioned, and the most illustrative result is shown in Figure 6. ANOVA showed a statistically significant effect (*α* = 0.05) of the important factors: the six molecular descriptor sets and the two machine learning algorithms.

For the mutagenicity dataset, the 2D descriptors delivered the best results: the scaled performance parameters are high, and those of the validation sets are in a narrow range. When examining the fingerprints, the best of them is the Atompair, which has high performance values for both algorithms. MACCS presents low classification values and a wide range of the validation sets. The two applied algorithms behave simultaneously, and only the XGBoost has somewhat higher scaled performance values, again. The range of the validation sets’ values remained in a narrow range for all descriptors and algorithms, and they overlapped almost in every case, except for the aforementioned MACCS, in which the range becomes relatively wide. We can state that the best and optimal classification results are to be acquired using the 2D descriptors.

### Results of the P-Glycoprotein Dataset

**FIGURE 7:**
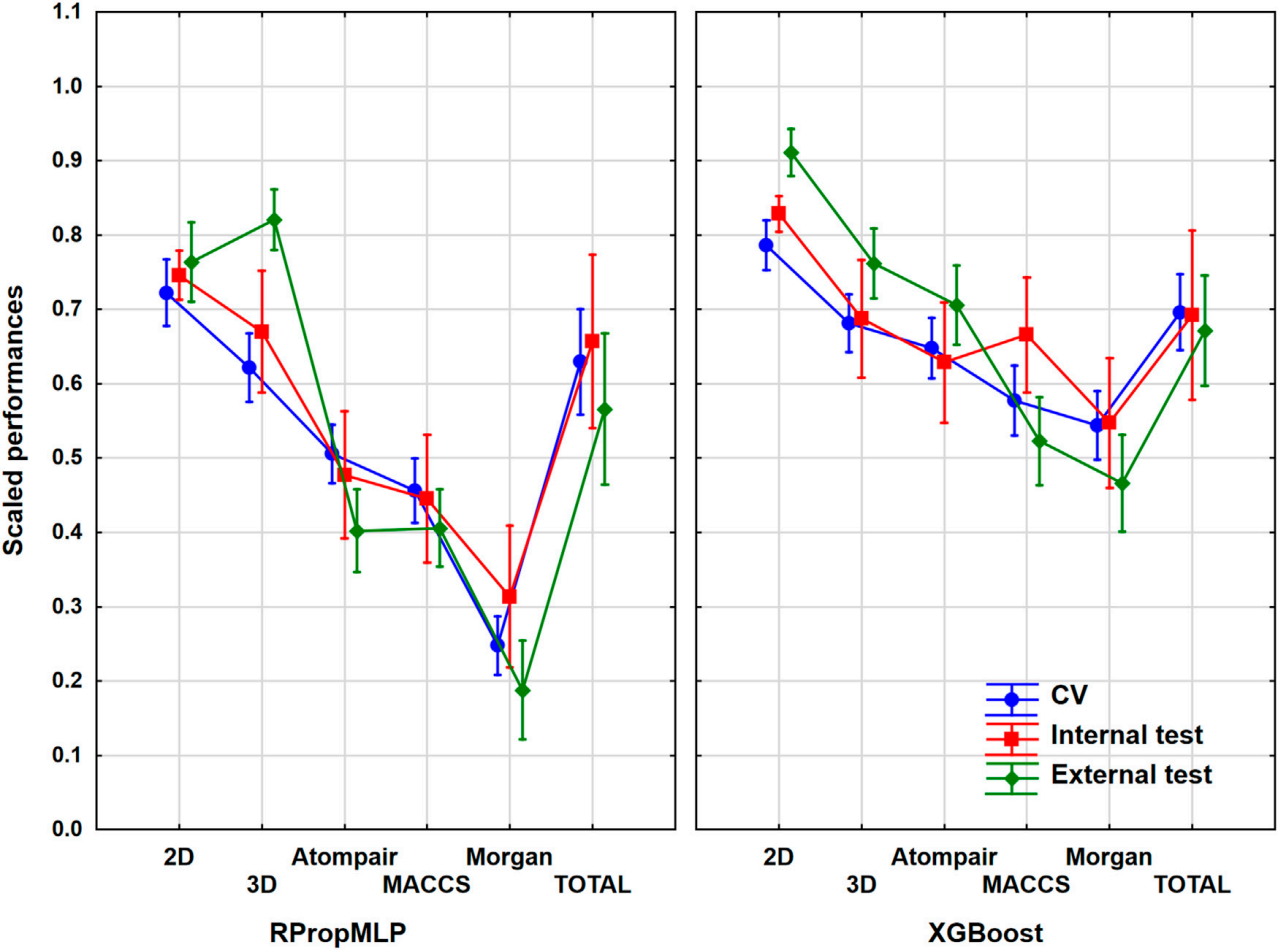
Scaled performances of the models in the case of P-glycoprotein dataset based on the molecular descriptor types and machine learning algorithms. The 18 performance parameters were range-scaled between 0 and 1 and averaged across all models with the given descriptor types/ML algorithms. The result of CV is marked with blue, the internal test result is marked with red, and the external test is marked with green. The mean of the scaled performance parameters is plotted in each case with the 95% confidence intervals.

The P-glycoprotein inhibition dataset was the smallest one amongst the six case studies, but it helped to see whether the examined patterns change with the use of smaller or larger datasets. It contained slightly more than 1,000 diverse molecules. ANOVA showed that still the effect of the molecular descriptor sets and the machine learning algorithms is significant. Figure 7 presents the illustrative result of the ANOVA.

The application of the 2D descriptors resulted in the best scaled performance parameters for both algorithms in the case of the P-gp dataset. As for the fingerprint descriptors, the best results were given by the Atompair for both algorithms. The two algorithms produced similar results for the 2D descriptors; however, for the rest of the descriptors, the XGBoost outperformed the RPropMLP. The external validation set resulted in the highest scaled performance values in case of the 2D and 3D descriptors combined with the RPropMLP and of the 2D, 3D, and Atompair for the XGBoost. The performance of the three validation sets fell in a somewhat narrow range except with the 3D descriptor for the RPropMLP. All in all, the three validation sets overlapped almost in every case. Finally, we can conclude that the best scaled performance values can be obtained employing the 2D descriptor set in combination with the XGBoost algorithm.

### Evaluation of the Datasets Together

**FIGURE 8:**
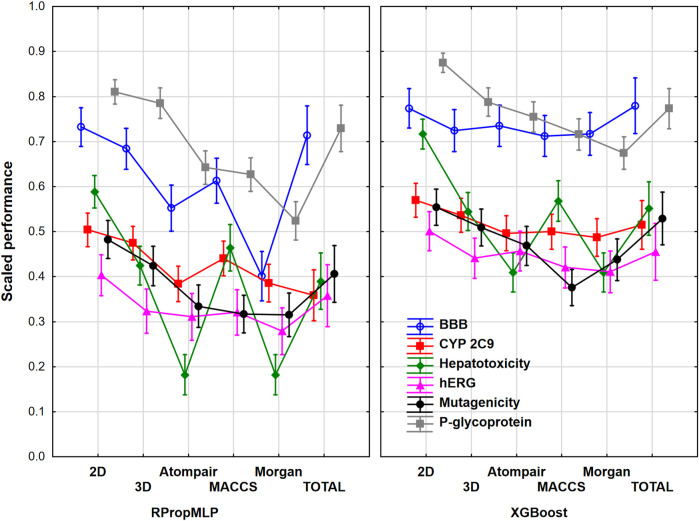
Scaled performances based on machine learning algorithms and molecular descriptor sets for all the six datasets. The 18 performance parameters were range-scaled between 0 and 1 and averaged across all models with the given descriptor types/ML algorithms/datasets. The case studies are plotted with different colors. Mean of the scaled performance parameters and the 95% confidence intervals are presented for each case.

We have examined the six case studies together to obtain general conclusions about the molecular descriptor sets and machine learning algorithms. For this purpose, we have range-scaled the performance parameters of the different models for all the six datasets together; thus, we could see the differences between the performance of the case studies, and it was necessary to eliminate the confusion of the scaled performance parameters, namely, the same scaled value could correspond to different original performance values in two datasets. At first, the molecular descriptor sets, the machine learning algorithms, and the different case studies were used as factors in ANOVA. The effect of these factors was statistically significant (*α* = 0.05), which means that significant differences can be detected between the groups in each factor. Figure 8 shows the plot of the three factors together for the united datasets.

**FIGURE 9:**
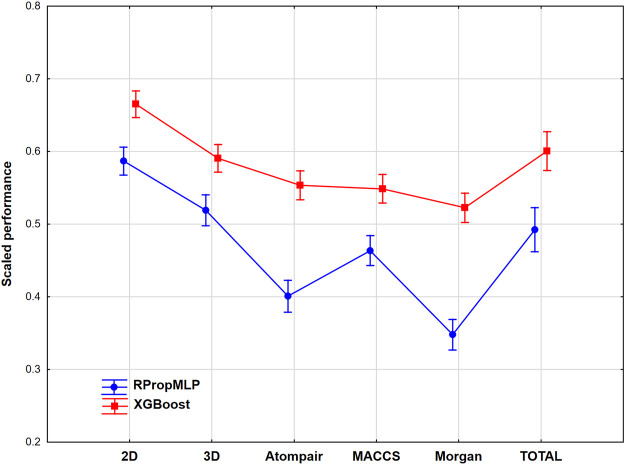
Scaled performance parameters are plotted based on molecular descriptors and machine learning methods. The 18 performance parameters were range-scaled between 0 and 1 and averaged across all models with the given descriptor types/ML algorithms. Mean of the scaled performance parameters and the 95% confidence intervals are presented for each case.

The 18 performance parameters were range-scaled between 0 and 1. This means that the differences between the performances of the datasets are valid, but the actual performance such as accuracy will be discussed later. The figure shows obvious differences between the neural network-based RPropMLP and the XGBoost algorithms: XGBoost worked better in all of the six case studies. The hepatotoxicity dataset had the most diverse results with the different molecular descriptor sets, while the hERG case study showed the least differences between the applied molecular descriptor sets. Still, it can be concluded that the use of 0-, 1-, and 2D descriptors (2D in the plot) can give the best models in all six case studies, which is closely followed using 3D descriptors alone and the use of all the descriptor sets together (total). The only exception was the hepatotoxicity dataset, in which the use of MACCS fingerprint was an equally good choice as the 3D or the total version. Out of the three fingerprint types, MACCS usually worked better than the Morgan and the Atompair fingerprints. This is clearly shown in Figure 9, which summarizes the findings based on ANOVA focusing only on the machine learning algorithms and the molecular descriptors as factors.

If we merge all the models together for the six case studies, we can conclude that the XGBoost algorithm can clearly outperform the RPropMLP method in the examined case studies. Although the differences are smaller in the case of the XGBoost method, still significant differences can be detected between the molecular descriptor sets. In the case of the XGBoost algorithm, 2D (0D, 1D, and 2D together) descriptors can be recommended instead of 3D descriptors, and it is worth checking it before we use combined descriptors. The same conclusion can be true for the neural network-based RPropMLP algorithm, but here even the application of the 3D descriptors is solely more effective than the use of the combined descriptors (total).

## Discussion

Based on the six case studies, we can support our previous analysis of the ADME-Tox-related classification models (Rácz et al., 2021). Here, we have found that the XGBoost algorithm could outperform the neural network-based models, which is consistent with the findings in the mentioned publication: the tree-based algorithms have high priority over the others in the ADME-Tox-related models from the literature of the last 5 years. However, the RPropMLP algorithm may provide better accuracies, if we optimized the model-building phase, but the tendencies between the applied descriptors are expected to remain the same. Also, while in the last few years, the combined descriptors (fingerprints with 2D or 3D descriptors) can be considered the most popular choice, based on our findings, it could be beneficial to use them separately and select the optimal ones before combining them. It is a general belief that the increase of the dimension of descriptors gives models with better predictive performances; however, it is not generally true (Doweyko, 2004).

**FIGURE 10:**
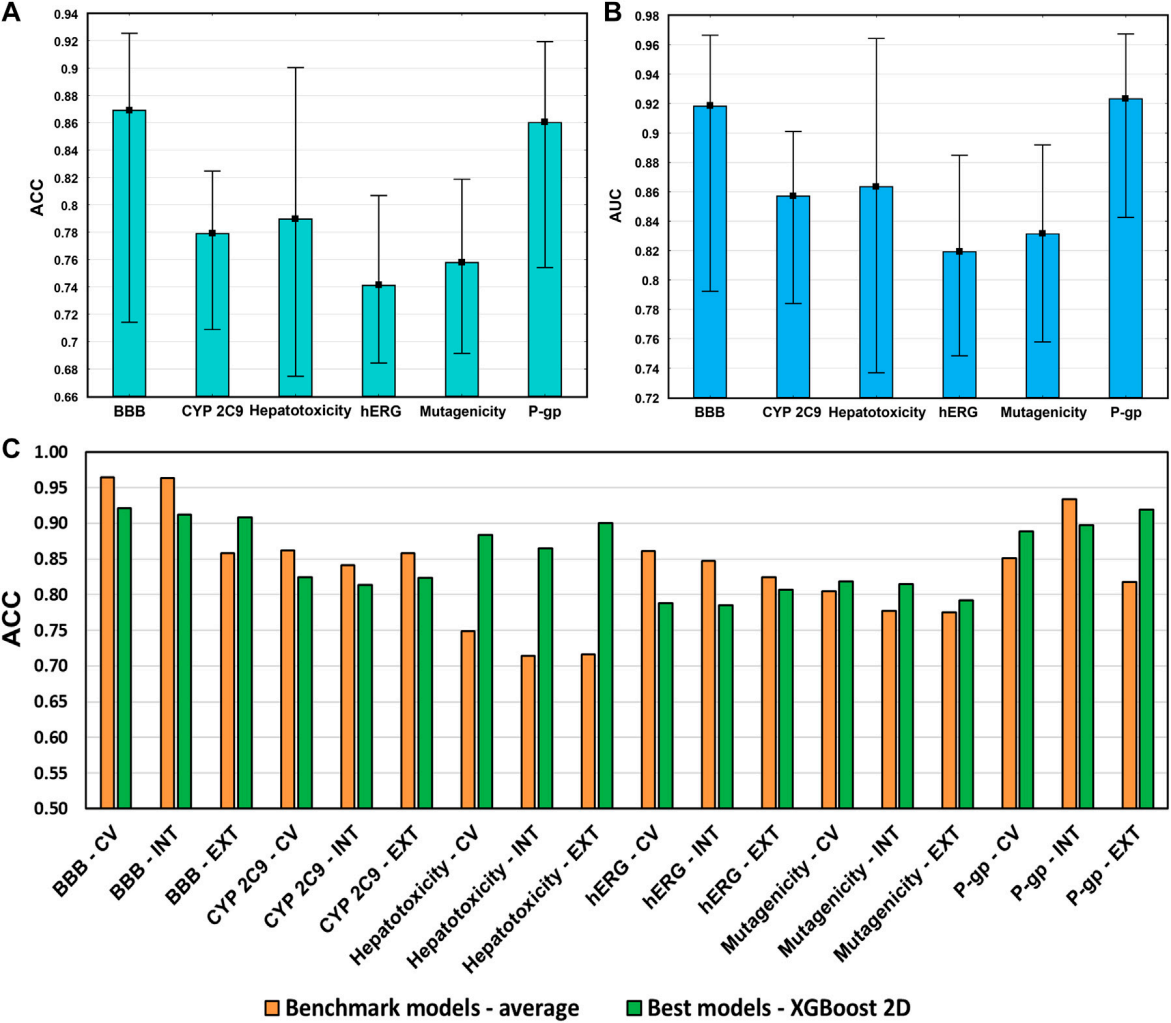
**(A)** Average accuracies (ACC) and **(B)** AUC values of the developed models in each case study. Minimum and maximum values are plotted together with the averages. **(C)** Comparison of the best models in each case study with the average performance of the literature data.

The average accuracies and AUC values of the models are shown in Figure 10 for each case study, with the min–max values. This way we could compare the models with the literature ones fairly because a complete validation of the benchmark models is usually missing. The best benchmark models cannot be determined based on solely one criterion. According to the literature data in our review (Rácz et al., 2021), the present models fit into the average performance in each case study. Moreover, Figure 10C shows that the best of the developed models sometimes can be even better. It means that our models were appropriate for further evaluations and to make our conclusions about the descriptor sets.

Our models were properly validated with cross-validation, internal validation, and external validation steps as well, which is highly recommended (even by the OECD guidelines), but still, it is not as popular in the literature as it should be. In this study, our primary intention was not to outperform the models in the literature for the selected case studies but to show the importance of the molecular descriptor set selection and its impact on the final models. Our findings give an indication about how to start making a good model, not how to improve it in a later phase. The result shows that the combination of the five kinds of descriptors does not improve the models sufficiently. It can increase the noise unless it is performed after a specific analysis of the data. As nowadays, computational capacities are capable of calculating hundreds and thousands of models, we tend to use as many input data as possible, but it is worth keeping in mind that sometimes *less can be more*.

## Conclusion

ADME-Tox QSPR modeling is an important aspect of the drug design process. Here, six popular ADME-Tox targets were presented with datasets over 1,000 diverse molecules for QSPR modeling. Our aim was to examine the effects of the molecular descriptor sets, namely, the 0–2D molecular descriptors and 3D descriptors and the Atompair, Morgan, and MACCS fingerprints on the performance of the binary classification models with two popular machine learning algorithms. The combination of the five descriptor types was also tested in the modeling phase. The applied 18 performance parameters of the developed models were appropriate for further evaluation. Factorial ANOVA showed the superiority of the XGBoost algorithm over the RPropMLP (neural network-based) algorithm in all cases. The results showed that the classical descriptor sets (especially the 0–2D descriptor set) can outperform the use of fingerprints alone. Although the combined set, in which all the five different descriptor sets were merged, can perform better than the fingerprint-based models, it could not outperform the classical 0–2D descriptor–based models for the examined ADME-Tox targets.

## Data Availability

The original contributions presented in the study are included in the article/Supplementary Material, further inquiries can be directed to the corresponding author.

## References

[B1] Abdelaziz A. Spahn-Langguth H. Schramm K.-W. Tetko I. V. (2016). Consensus Modeling for HTS Assays Using In Silico Descriptors Calculates the Best Balanced Accuracy in Tox21 Challenge. Front. Environ. Sci. 4, 2. 10.3389/fenvs.2016.00002

[B2] Alves V. M. Golbraikh A. Capuzzi S. J. Liu K. Lam W. I. Korn D. R. (2018). Multi-Descriptor Read across (MuDRA): A Simple and Transparent Approach for Developing Accurate Quantitative Structure-Activity Relationship Models. J. Chem. Inf. Model.. 58, 1214–1223. 10.1021/acs.jcim.8b00124 29809005 PMC7917006

[B3] Bajusz D. Rácz A. Héberger K. (2017). “Chemical Data Formats, Fingerprints, and Other Molecular Descriptions for Database Analysis and Searching,” in Comprehensive Medicinal Chemistry III. Editors Chackalamannil S. Rotella D. P. Ward S. E. (Oxford: Elsevier), 329–378. 10.1016/B978-0-12-409547-2.12345-5

[B4] Basak S. C. Gute B. D. Grunwald G. D. (1997). Use of Topostructural, Topochemical, and Geometric Parameters in the Prediction of Vapor Pressure: A Hierarchical QSAR Approach. J. Chem. Inf. Comput. Sci. 37, 651–655. 10.1021/ci960176d

[B5] Broccatelli F. Carosati E. Neri A. Frosini M. Goracci L. Oprea T. I. (2011). A Novel Approach for Predicting P-Glycoprotein (ABCB1) Inhibition Using Molecular Interaction Fields. J. Med. Chem. 54, 1740–1751. 10.1021/jm101421d 21341745 PMC3069647

[B6] Carhart R. E. Smith D. H. Venkataraghavan R. (1985). Atom Pairs as Molecular Features in Structure-Activity Studies: Definition and Applications. J. Chem. Inf. Comput. Sci. 25, 64–73. 10.1021/ci00046a002

[B7] Chen T. Guestrin C. (2016). “XGBoost,” in Proceedings of the 22nd ACM SIGKDD International Conference on Knowledge Discovery and Data Mining (New York, NY, USA: ACM), 785–794. 10.1145/2939672.2939785

[B8] Danishuddin V. Kumar V. Faheem M. Woo Lee K. (2022). A Decade of Machine Learning-Based Predictive Models for Human Pharmacokinetics: Advances and Challenges. Drug Discov. Today 27, 529–537. 10.1016/j.drudis.2021.09.013 34592448

[B9] Davis A. P. Grondin C. J. Johnson R. J. Sciaky D. McMorran R. Wiegers J. (2019). The Comparative Toxicogenomics Database: Update 2019. Nucleic Acids Res. 47, D948–D954. 10.1093/nar/gky868 30247620 PMC6323936

[B10] Doweyko A. M. (2004). 3D-QSAR Illusions. J. Comput. Aided. Mol. Des. 18, 587–596. 10.1007/s10822-004-4068-0 15729857

[B11] Ferreira L. L. G. Andricopulo A. D. (2019). ADMET Modeling Approaches in Drug Discovery. Drug Discov. Today 24, 1157–1165. 10.1016/j.drudis.2019.03.015 30890362

[B12] Friedman J. H. (2001). Greedy Function Approximation: A Gradient Boosting Machine. Ann. Stat. 29, 1189–1232. 10.1214/aos/1013203451

[B13] Gramatica P. (2007). Principles of QSAR Models Validation: Internal and External. QSAR Comb. Sci. 26, 694–701. 10.1002/qsar.200610151

[B14] Gramatica P. Sangion A. (2016). A Historical Excursus on the Statistical Validation Parameters for QSAR Models: A Clarification Concerning Metrics and Terminology. J. Chem. Inf. Model. 56, 1127–1131. 10.1021/acs.jcim.6b00088 27218604

[B15] Hansen K. Mika S. Schroeter T. Sutter A. ter Laak A. Steger-Hartmann T. (2009). Benchmark Data Set for In Silico Prediction of Ames Mutagenicity. J. Chem. Inf. Model.. 49, 2077–2081. 10.1021/ci900161g 19702240

[B16] Kuhn M. Campillos M. Letunic I. Jensen L. J. Bork P. (2010). A Side Effect Resource to Capture Phenotypic Effects of Drugs. Mol. Syst. Biol. 6, 343. 10.1038/msb.2009.98 20087340 PMC2824526

[B17] Lei T. Li Y. Song Y. Li D. Sun H. Hou T. (2016). ADMET Evaluation in Drug Discovery: 15. Accurate Prediction of Rat Oral Acute Toxicity Using Relevance Vector Machine and Consensus Modeling. J. Cheminform. 8, 6. 10.1186/s13321-016-0117-7 26839598 PMC4736633

[B18] Lima A. N. Philot E. A. Trossini G. H. G. Scott L. P. B. Maltarollo V. G. Honorio K. M. (2016). Use of Machine Learning Approaches for Novel Drug Discovery. Expert Opin. Drug Discov. 11, 225–239. 10.1517/17460441.2016.1146250 26814169

[B19] Morgan H. L. (1965). The Generation of a Unique Machine Description for Chemical Structures-A Technique Developed at Chemical Abstracts Service. J. Chem. Doc. 5, 107–113. 10.1021/c160017a018

[B20] Nembri S. Grisoni F. Consonni V. Todeschini R. (2016). In Silico Prediction of Cytochrome P450-Drug Interaction: QSARs for CYP3A4 and CYP2C9. Ijms 17, 914. 10.3390/ijms17060914 PMC4926447 27294921

[B21] PubChem (2021). Cytochrome Panel Assay with Activity Outcomes. Natl. Cent. Biotechnol. Inf. Source=NCGC. Available at: https://pubchem.ncbi.nlm.nih.gov/bioassay/1851 (Accessed October 27, 2021).

[B22] Rácz A. Bajusz D. Héberger K. (2015). Consistency of QSAR Models: Correct Split of Training and Test Sets, Ranking of Models and Performance Parameters. Sar. QSAR Environ. Res. 26, 683–700. 10.1080/1062936X.2015.1084647 26434574

[B23] Rácz A. Bajusz D. Héberger K. (2019a). Intercorrelation Limits in Molecular Descriptor Preselection for QSAR/QSPR. Mol. Inf. 38, 1800154. 10.1002/minf.201800154 PMC6767540 30945814

[B24] Rácz A. Bajusz D. Héberger K. (2019b). Multi-Level Comparison of Machine Learning Classifiers and Their Performance Metrics. Molecules 24, 2811–2818. 10.3390/molecules24152811 PMC6695655 31374986

[B25] Rácz A. Bajusz D. Miranda-Quintana R. A. Héberger K. (2021). Machine Learning Models for Classification Tasks Related to Drug Safety. Mol. Divers. 25, 1409–1424. 10.1007/s11030-021-10239-x 34110577 PMC8342376

[B26] Rácz A. Keserű G. M. (2020). Large-scale Evaluation of Cytochrome P450 2C9 Mediated Drug Interaction Potential with Machine Learning-Based Consensus Modeling. J. Comput. Aided. Mol. Des. 34, 831–839. 10.1007/s10822-020-00308-y 32221780 PMC7320947

[B27] Ravi M. Hopfinger A. J. Hormann R. E. Dinan L. (2001). 4D-QSAR Analysis of a Set of Ecdysteroids and a Comparison to CoMFA Modeling. J. Chem. Inf. Comput. Sci. 41, 1587–1604. 10.1021/ci010076u 11749586

[B29] Riedmiller M. Braun H. (1993). A Direct Adaptive Method for Faster Backpropagation Learning: the RPROP Algorithm. IEEE Int. Conf. Neural Netw. 1, 586–591. 10.1109/ICNN.1993.298623

[B30] Roy D. Hinge V. K. Kovalenko A. (2019). To Pass or Not to Pass: Predicting the Blood-Brain Barrier Permeability with the 3D-RISM-KH Molecular Solvation Theory. ACS Omega 4, 16774–16780. 10.1021/acsomega.9b01512 31646222 PMC6796930

[B31] Schrödinger Release 2021-3 (2021). MacroModel. Schrödinger, LLC. New York, NY.

[B32] Sheridan R. P. Wang W. M. Liaw A. Ma J. Gifford E. M. (2016). Extreme Gradient Boosting as a Method for Quantitative Structure-Activity Relationships. J. Chem. Inf. Model.. 56, 2353–2360. 10.1021/acs.jcim.6b00591 27958738

[B33] Steinbeck C. Han Y. Kuhn S. Horlacher O. Luttmann E. Willighagen E. (2003). The Chemistry Development Kit (CDK): An Open-Source Java Library for Chemo- and Bioinformatics. J. Chem. Inf. Comput. Sci. 43, 493–500. 10.1021/ci025584y 12653513 PMC4901983

[B34] Tatonetti N. P. Ye P. P. Daneshjou R. Altman R. B. (2012). Data-Driven Prediction of Drug Effects and Interactions. Sci. Transl. Med. 4, 377. 10.1126/scitranslmed.3003377 PMC3382018 22422992

[B35] Todeschini R. Consonni V. (2000). Handbook of Molecular Descriptors. Weinheim, Germany: Wiley VCH.

[B36] Tsou L. K. Yeh S.-H. Ueng S.-H. Chang C.-P. Song J.-S. Wu M.-H. (2020). Comparative Study between Deep Learning and QSAR Classifications for TNBC Inhibitors and Novel GPCR Agonist Discovery. Sci. Rep. 10, 16771. 10.1038/s41598-020-73681-1 33033310 PMC7545175

[B37] Wang L. Pang X. Li Y. Zhang Z. Tan W. (2016). RADER: a RApid DEcoy Retriever to Facilitate Decoy Based Assessment of Virtual Screening. Bioinformatics, btw783. 10.1093/bioinformatics/btw783 28011765

[B38] Wu Q. Cai C. Guo P. Chen M. Wu X. Zhou J. (2019). In Silico Identification and Mechanism Exploration of Hepatotoxic Ingredients in Traditional Chinese Medicine. Front. Pharmacol. 10, 1–15. 10.3389/fphar.2019.00458 31130860 PMC6509242

[B39] Yang H. Sun L. Li W. Liu G. Tang Y. (2018). In Silico Prediction of Chemical Toxicity for Drug Design Using Machine Learning Methods and Structural Alerts. Front. Chem. 6, 1–12. 10.3389/fchem.2018.00030 29515993 PMC5826228

[B40] Zhang D. Gong Y. (2020). The Comparison of LightGBM and XGBoost Coupling Factor Analysis and Prediagnosis of Acute Liver Failure. IEEE Access 8, 220990–221003. 10.1109/ACCESS.2020.3042848

