# Supplementary figures and images for: Comparison of Descriptor- and Fingerprint Sets in Machine Learning Models for ADME-Tox Targets
Source: Front Chem. 2022 Jun 8;10:852893. doi: 10.3389/fchem.2022.852893 (PMC9214226; doi:10.3389/fchem.2022.852893)

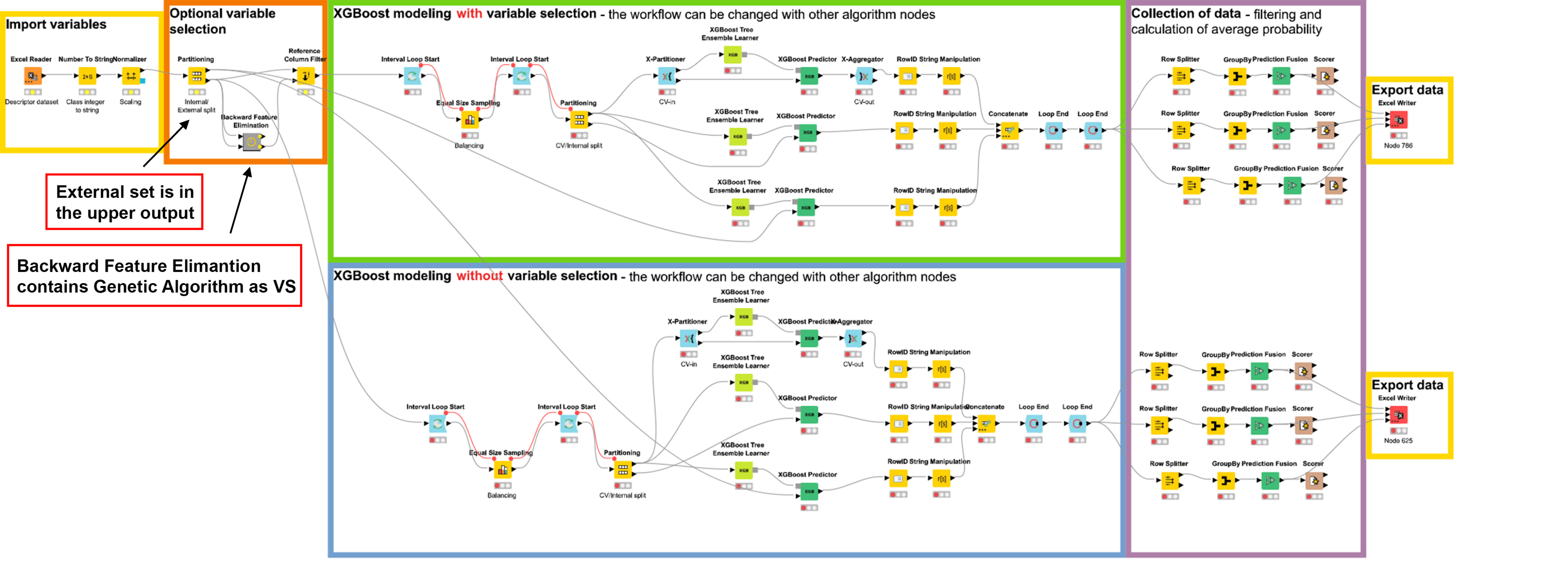

Supplement: Supplementary file 1 [file Image1.TIF]
