# Supplementary material for: Comparison of Descriptor- and Fingerprint Sets in Machine Learning Models for ADME-Tox Targets
Source: Front Chem. 2022 Jun 8;10:852893. doi: 10.3389/fchem.2022.852893 (PMC9214226; doi:10.3389/fchem.2022.852893)
Supplement: Supplementary file 2 [file DataSheet1.docx]

**Supplementary material**

**Comparison of descriptor- and fingerprint sets in machine learning models for ADME-Tox targets**

Álmos Orosz^1,2^, Károly Héberger^1,*^, Anita Rácz^1^

^1^ Plasma Chemistry Research Group, Research Centre for Natural Sciences, Magyar tudósok krt. 2, 1117 Budapest, Hungary

*corresponding author: Károly Héberger, heberger.karoly@ttk.hu

**Table S1.** The number of the original input variables for each dataset in the different molecular descriptor sets.

| **Dataset** | **2D** | **3D** | **Atompair** | **MACCS** | **ECFP** |
| --- | --- | --- | --- | --- | --- |
| **BBB** | **1843** | **1168** | **1024** | **166** | **1024** |
| **CYP 2C9** | **2328** | **1233** | **1024** | **166** | **1024** |
| **Hepatotoxicity** | **2039** | **1179** | **1024** | **166** | **1024** |
| **hERG** | **1941** | **1207** | **1024** | **166** | **1024** |
| **P-gp** | **1813** | **1161** | **1024** | **166** | **1024** |
| **Mutagenicity** | **2075** | **1199** | **1024** | **166** | **1024** |

**Table S2.** The applied 18 performance parameters for the determination of the goodness of the models. Equations can be found in our previous work (Rácz et al., 2019).

| **AUAC** | Area under the accumulation curve |
| --- | --- |
| **AUC** | Area under the ROC curve |
| **AP** | Average precision |
| **TPR** | True positive rate (TPR), sensitivity |
| **TNR** | True negative rate, specificity |
| **PPV** | Positive predictive value, precision |
| **NPV** | Negative predictive value |
| **BM** | Bookmaker informedness |
| **MK** | Markedness |
| **LRp** | Positive likelihood ratio |
| **LRn** | Negative likelihood ratio |
| **DOR** | Diagnostic odds ratio |
| **MCC** | Matthews correlation coefficient |
| **Cohen κ** | Cohen’s kappa |
| **ACC** | Accuracy, correct classification rate |
| **BACC** | Balanced accuracy |
| **Jaccard** | Jaccard score |
| **F1** | F1 score, F measure |

**Figure S1.** *t*-SNE plots for each dataset, internal and external sets separately. Internal sets are marked with red, external sets are marked with blue. A) BBB, B) CYP 2C9, C) Hepatotoxicity, D) hERG, E) Mutagenicity and F) P-glycoprotein.


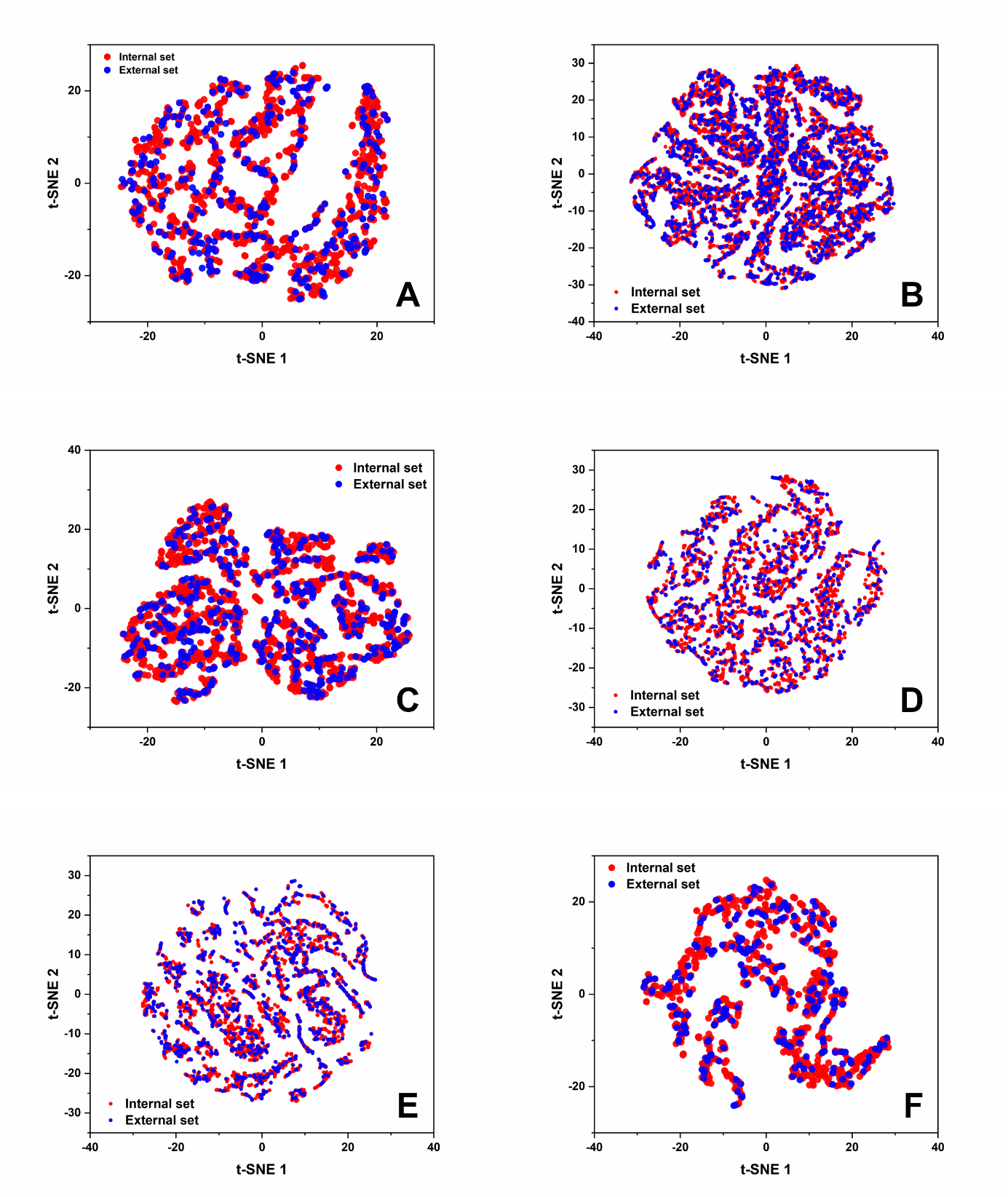


**References**

Rácz, A., Bajusz, D., and Héberger, K. (2019). Multi-Level Comparison of Machine Learning Classifiers and Their Performance Metrics. *Molecules* 24. doi:10.3390/molecules24152811.
